# Mechanisms and Regulation of Cardiac Ca_V_1.2 Trafficking

**DOI:** 10.3390/ijms22115927

**Published:** 2021-05-31

**Authors:** Maartje Westhoff, Rose E. Dixon

**Affiliations:** Department of Physiology and Membrane Biology, School of Medicine, University of California, Davis, CA 95616, USA; mfwesthoff@ucdavis.edu

**Keywords:** L-type calcium channels, ion channel trafficking, t-tubule, caveolae, calcium signaling, β-adrenergic receptor, angiotensin II

## Abstract

During cardiac excitation contraction coupling, the arrival of an action potential at the ventricular myocardium triggers voltage-dependent L-type Ca^2+^ (Ca_V_1.2) channels in individual myocytes to open briefly. The level of this Ca^2+^ influx tunes the amplitude of Ca^2+^-induced Ca^2+^ release from ryanodine receptors (RyR2) on the junctional sarcoplasmic reticulum and thus the magnitude of the elevation in intracellular Ca^2+^ concentration and ultimately the downstream contraction. The number and activity of functional Ca_V_1.2 channels at the t-tubule dyads dictates the amplitude of the Ca^2+^ influx. Trafficking of these channels and their auxiliary subunits to the cell surface is thus tightly controlled and regulated to ensure adequate sarcolemmal expression to sustain this critical process. To that end, recent discoveries have revealed the existence of internal reservoirs of preformed Ca_V_1.2 channels that can be rapidly mobilized to enhance sarcolemmal expression in times of acute stress when hemodynamic and metabolic demand increases. In this review, we provide an overview of the current thinking on Ca_V_1.2 channel trafficking dynamics in the heart. We highlight the numerous points of control including the biosynthetic pathway, the endosomal recycling pathway, ubiquitination, and lysosomal and proteasomal degradation pathways, and discuss the effects of β-adrenergic and angiotensin receptor signaling cascades on this process.

## 1. Introduction

Voltage-gated, L-type Ca_V_1.2 channels play an essential role in cardiac excitation-contraction (EC) coupling and can regulate cardiac gene expression. The number and activity of voltage-gated, L-type Ca_V_1.2 channels localized to specialized dyadic regions of the t-tubule sarcolemma, adjacent to ryanodine receptor (RyR2) clusters, dictates the degree of Ca^2+^ influx into cardiomyocytes and is thus a major determinant of the magnitude of ventricular contraction. On the other hand, Ca_V_1.2 channels localized to caveolae are thought to play a critical role in regulation of gene expression in a process known as excitation–transcription coupling. Targeting of Ca_V_1.2 channels to the appropriate membrane compartment is thus critical for their proper physiological function. The number of Ca_V_1.2 channels at the sarcolemma at any given time is governed by the relative amount of channel insertions achieved with anterograde trafficking via the secretory and recycling pathways, versus endocytosis via retrograde trafficking pathways. Endocytosed channels can refuel recycling endosome pathways to provide a rapidly mobilizable pool of channels, or they can be targeted for degradation in lysosomes or the proteasome. In this review, we summarize the current literature on Ca_V_1.2 channel trafficking and its regulation. We highlight recent data indicating that G-protein coupled receptor (GPCR)-signaling can positively (in the case of β-adrenergic receptors) or negatively (in the case of Angiotensin type 1 receptors; AT_1_R) influence the surface abundance of Ca_V_1.2 channels, providing a means to tune cardiac EC-coupling by altering channel expression. We begin with a brief summary of the structure and function of these multimeric channels.

## 2. Ca_V_1.2 Channel Structure and Function

### 2.1. Ca_V_α Subunits

**Figure 1:**
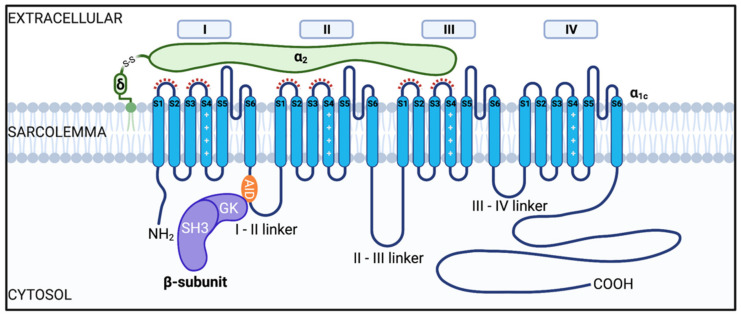
Illustration of the cardiac voltage gated L-type Ca_V_1.2 channel complex. The channel consists of a pore-forming Ca_V_α_1C_ subunit and auxiliary subunits Ca_V_β and Ca_V_α_2_δ. The Ca_V_α_1c_ is composed of four homologous repeat domains (I-IV), each having six transmembrane spanning segments (S1–S6, shown in blue). S1–S4 comprise the voltage sensing domain (VSD) and S5–S6 form the pore domain (PD). Ca_V_β subunits (depicted in purple) are composed of a SH3, HOOK, and GK domain. Interaction of Ca_V_β with the Ca_V_α_1_c occurs between the GK domain on Ca_V_β subunits and the alpha interaction domain (AID) on the I-II linker (orange). Ca_V_α_2_δ subunits are proposed to interact with the extracellular loops of domains I-III as highlighted by the red dashed lines [10,11,12,13].

L-type Ca^2+^ channels (LTCC) are a family of voltage-gated Ca^2+^ channels (Ca_V_1.1–1.4) that allow Ca^2+^ influx into excitable cells in response to depolarization. Their structure and function is reviewed extensively elsewhere [1,2,3]. Here we focus on trafficking and regulation of Ca_V_1.2, a multimeric protein complex composed of a pore-forming α_1_ subunit encoded by cacna1c, and associated auxiliary subunits Ca_V_β, Ca_V_α_2_δ and sometimes, Ca_V_γ [1]. Ca_V_α_1C_ subunits are widely expressed in brain, smooth muscle, and pancreas, and they form the core of the most prevalent L-type Ca^2+^ channel in the cardiac muscle of the heart [4,5]. Underscoring their fundamental importance in cardiac function, cacna1c knockout is embryonic lethal in mice [6]. The typical structure of the Ca_V_1.2 channel complex is depicted in Figure 1. When expressed alone, Ca_V_α_1C_ subunits are not efficiently inserted into the membrane resulting in very low current density [7,8,9]. The full functional identity of the channel becomes evident once associated with its auxiliary subunits.

### 2.2. Ca_V_β Subunits

Ca_V_β subunits are cytosolic proteins with Src homology 3 (SH3), HOOK, and guanylate kinase (GK) domains forming the core of the protein responsible for the majority of functional properties of the β subunits [14,15,16,17]. There are four main Ca_V_β subunits (Ca_V_β1-4) encoded by separate genes with several splice variants. Ca_V_β_2b_ is the most highly expressed isoform in the heart [9]. Accordingly, Ca_V_β_2_^−/−^ mice suffer embryonic lethality and disrupted cardiac phenotype [18]. In heterologous and native systems interactions between Ca_V_β and Ca_V_α_1c_ subunits lead to alterations in channel activation and inactivation as well as robust increases in surface expression and current density, likely due to an increased open probability and/or enhanced cell membrane localization of the channel complex [9,19,20,21,22,23]. This 1:1 stochiometric interaction between the subunits is thought to occur within the α-interaction domain (AID), located within the I-II pore loop of Ca_V_α_1c_ subunits [21,24], and small region of the GK domain on Ca_V_β, referred to as the AID-binding pocket (ABP) [16]. Mutations within this region interfere with Ca_V_β and Ca_V_α_1c_ interactions and result in diminished membrane targeting of Ca_V_α_1c_ [21]. Furthermore, deletion mutants of regions within the Ca_V_α_1c_ C-terminus result in reduced plasma membrane localized channels, with many channels remaining stuck in internal membranes [25].

### 2.3. Ca_V_α_2_δ Subunits

Ca_V_α_2_δ, is comprised of two subunits α_2_ and δ which are encoded by the same gene and bound together by disulfide bonds. The N-terminal α_2_ region is extracellular, with the δ region anchored to the outer leaflet of the plasma membrane via a GPI anchor [26]. Of the several isoforms, Ca_V_α_2_δ1-3 have been found in the heart [27,28]. Within all the isoforms there is a Von Willebrand factor-A (VWA) domain in the extracellular region, and in Ca_V_α_2_δ_1_ and Ca_V_α_2_δ_2_ this consists of a metal-ion-dependent adhesion site (MIDAS) which is thought to interact directly with the extracellular loops of Ca_V_α_1c_ [11,29]. When this MIDAS site is mutated, there is a diminished trafficking response and retention of the subunits within the ER [29]. As well as promoting trafficking of the channel complex, association of Ca_V_α_2_δ with Ca_V_α_1c_ also enhances the voltage-dependent activation by increasing the voltage sensitivity of the VSDs, allowing for calcium influx at more physiological membrane potentials [10].

### 2.4. Ca_V_γ Subunits

The final auxiliary subunit, γ, is composed of four transmembrane spanning segments with intracellular N- and C-termini. This is the least studied auxiliary subunit of cardiac Ca_V_1.2 channels but of the eight isoforms of γ subunits, four have been shown to be expressed in cardiac tissue and to associate with the Ca_V_1.2 complex (Ca_V_γ4, Ca_V_γ6, Ca_V_γ7 and Ca_V_γ8). In HEK293 cells coexpression of Ca_V_α_1c_ with the various Ca_V_γ subunits results in altered activation and inactivation kinetics of the whole cell Ca^2+^ current (*I*_Ca_) [30]. However, the specific role of endogenous Ca_V_γ subunits in native heart tissue and their effects on channel trafficking remain unknown.

## 3. Trafficking of Ca_V_1.2 Channels

### 3.1. Anterograde Transport of Ca_V_1.2 to the Sarcolemma

**Figure 2:**
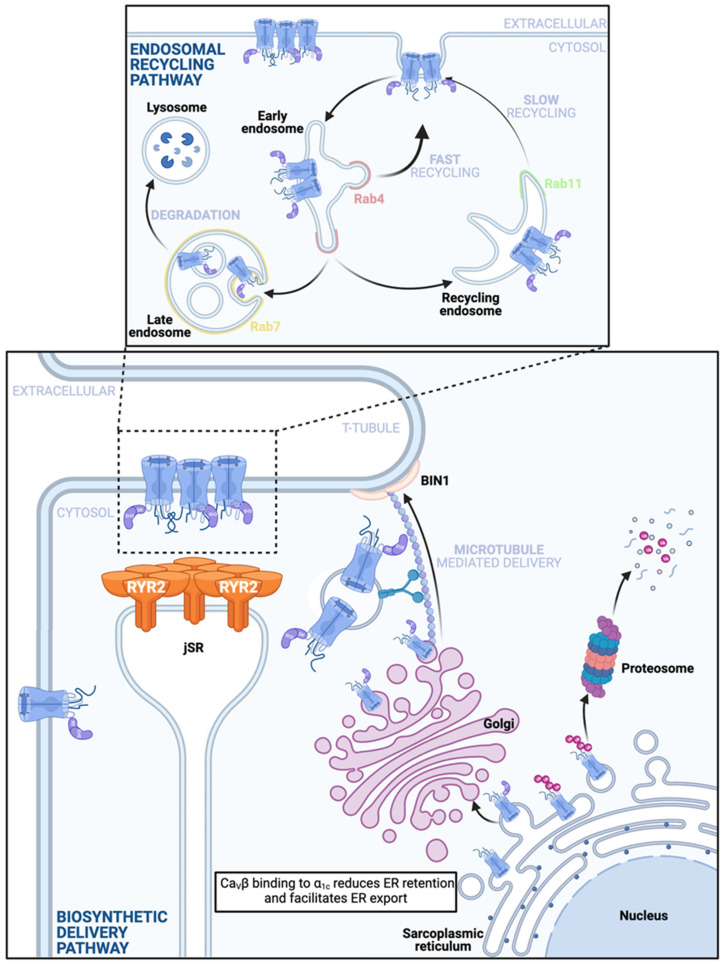
Anterograde Ca_V_1.2 channel delivery pathways. Bottom: A depiction of the ‘Biosynthetic Delivery Pathway’ that commences when Ca_V_α_1c_ are translated directly into the rER membrane. These pore-forming subunits have several ER retention motifs and just one identified ER export motif. Binding of the Ca_V_β subunit to the Ca_V_α_1c_ while in the ER membrane is thought to lessen the influence of the retention signals and favor channel export, whereby the channels subsequently exit the ER in vesicles to the Golgi complex. Absence of the Ca_V_β subunit may render the channels more vulnerable to ubiquitination on the ER membrane, precipitating proteosomal degradation. Note this vulnerability has been reported in neuronal Ca_V_1.2 and has not yet been confirmed in the cardiac channels. Single or clustered channels exit the *trans*-Golgi network (TGN) and are carried along microtubules by motor proteins to BIN1-anchored delivery hubs on the t-tubule sarcolemma to assume positions in dyadic and caveolar regions. It is unclear exactly when and where in the anterograde trafficking pathway Ca_V_α_2_δ joins the complex. Top: Stable sarcolemmal Ca_V_1.2 channel expression is maintained in the face of ongoing channel internalization by the endosomal recycling pathway. Endocytosed channels enter the early endosome and are sorted, a subpopulation is sent to Rab7 positive late endosomes and may subsequently undergo lysosomal degradation. The remainder of the channels are recycled back to the membrane either through a fast, direct pathway from the early endosome along a Rab4-dependent fast recycling pathway, or through a slower Rab11-dependent recycling pathway from the recycling endosome. If some channels are degraded, it is possible that endosomal reservoirs of channels take their place and supplement this pathway to maintain stable membrane expression.

Ca_V_α_1c_, like all proteins destined for the cell membrane, is synthesized by membrane-bound ribosomes on the rough endoplasmic reticulum (rER) [31]. This is the first step in the classical secretory pathway. Briefly, free ribosomes initially pick-up the mRNA for the channel in the cytosol and begin translation but this is paused once the ribosome translates a signal sequence, A.K.A. the leader sequence. This is a short, ~20 amino acid long chain of hydrophobic amino acids near the N-terminus of the polypeptide, that once translated, finds and binds a signal recognition particle (SRP) that initiates targeting of the entire complex to the rER membrane. This idea was originally proposed by Blobel and Sabatini in their ‘signal hypothesis’ [32]. Recognition and binding of the signal sequence and ribosome by the SRP puts a halt to translation until the SRP binds to an SRP receptor. Once situated on the rER membrane, the SRP is released and the ribosome and polypeptide are handed off to a protein translocation complex (Sec61) where translation resumes. The Sec61 translocon complex forms an ER membrane spanning channel that acts as a conduit for entry of the growing polypeptide chain into the ER [33]. Since Ca_V_α_1c_ is a transmembrane protein bound for the plasma membrane, it is directly inserted into the ER membrane as it forms, with the N and C-termini located within the cytosol, the extracellular loops in the ER lumen, and the hydrophobic transmembrane regions spanning the ER membrane. Ca_V_α_2_δ and Ca_V_γ are similarly produced while cytosolic proteins like Ca_V_β subunits lack signal sequences and are thus translated on free ribosomes and released directly into the cytosol. Whether the entire channel complex assembles in the ER membrane remains unclear but there is a school of thought suggesting that binding of at least Ca_V_β to Ca_V_α_1c_ is necessary to release the channels from the ER and promote forward trafficking (discussed in more detail below). Upon their exit, the channels move in vesicles onwards to the Golgi apparatus, subsequently exiting from *trans*-Golgi complex in vesicles that are propelled by kinesin motor proteins along microtubule highways to their t-tubular, caveolar, or surface sarcolemmal destinations (see Figure 2).

### 3.2. Role of the Ca_V_β-Subunit in Ca_V_1.2 Trafficking

Until very recently, the prevailing thought was that Ca_V_β subunit interactions with α_1c_ were indispensable for channel trafficking to the cardiomyocyte surface membrane where the channels fulfill a duality of functions at the t-tubules in triggering Ca^2+^ induced Ca^2+^ release from RyR2, and contributing to loading of the SR [34], and play a further role at the caveolae in activation of calcineurin-NFAT signaling pathways [35]. The role of the Ca_V_β-subunit in membrane targeting of the channels has been extensively reviewed elsewhere [36,37] and so we mention it only briefly here to highlight some more recent advances [38,39]. In heterologous expression systems and in neurons, interactions between β-subunits and α_1_ are fundamentally required for channel trafficking to the plasma membrane [7,19,24,40,41,42,43,44]. Expression of the α_1_-subunit alone in the absence of the β-subunit, yields dramatically reduced surface expression and little to no currents, in contrast to the robust surface expression and currents in cells in which the β-subunit is co-expressed [19,23,43,45,46,47]. This effect is abrogated by mutations in the AID or the ABP suggesting that it requires Ca_V_β binding to the AID on the I-II loop of Ca_V_α_1_ [48,49,50]. It was initially suggested that this interaction shielded an ER retention motif on the I-II loop and thus promoted enhanced exit of the channel from the ER and on through the anterograde trafficking pathway to the surface membrane [51]. However, this idea was later refuted in work from the Colecraft lab that used chimeric channel constructs in which various intracellular portions of Ca_V_α_1c_ were substituted into another calcium channel (the α_1g_ of Ca_V_3.1) known to exhibit Ca_V_β-independent surface expression [23]. In this paradigm-shifting study, it was revealed that the I-II loop actually contains an ER-export signal whereas all the other intracellular loops and the N- and C-termini contain ER-retention signals. It is thus thought that binding of the Ca_V_β to the AID induces a conformational change in the channel that weakens the ER retention influences and instead shifts the balance toward ER export and trafficking to the surface. Furthermore, in heterologous expression systems and hippocampal neurons, binding of Ca_V_β to Ca_V_α_1c_ in the ER, reportedly protects the channel from ubiquitination and proteosomal degradation [52].

In cardiomyocytes the cellular architecture is vastly different from that of heterologous cells, but until recently, the same principals and reliance on the Ca_V_β-subunit for surface trafficking were thought to apply, in part fueled by studies showing that short hairpin RNA-mediated knockdown of Ca_V_β_2_ reduced *I*_Ca_ in adult rat ventricular myocytes by ~60% [53], and others showing that Ca_V_β_2_^−/−^ mice succumbed to embryonic lethality at around E10.5 due to heart failure caused by reduced L-type calcium current [18]. Marx and colleagues have cast doubt on the reliance of cardiac Ca_V_α_1c_ on Ca_V_β for trafficking in a study where they generated transgenic mice that expressed WT Ca_V_α_1c_ and could be doxycycline-induced to additionally express dihydropyridine (DHP)-resistant Ca_V_α_1c_ with a triple alanine mutation in their AID that significantly decreases the affinity of Ca_V_β-binding [44]. The AID mutant channels did not co-immunoprecipitate (co-IP) with Ca_V_β but did display t-tubule localization and generated whole-cell Ca^2+^ currents that could be distinguished from WT channels with addition of the DHP nisoldipine. This study raises questions about the role of Ca_V_β in Ca_V_α_1c_ trafficking in cardiomyocytes, however, the interpretation of these results is complicated by the presence of WT channels. Work from our lab reveals that Ca_V_1.2 channels often insert into the sarcolemma as large, multi-channel clusters [54]. In addition, we and others have shown that Ca_V_1 channels can functionally interact within clusters, such that the conformational change in one channel that occurs upon gating, can be transmitted to adjacent interacting channels, triggering their coordinate opening [55,56,57,58,59,60,61,62,63,64]. With this in mind, it seems possible that the conformational change induced by Ca_V_β subunits binding to WT channels, could be conferred to β-less AID mutant channels in the cluster and that this allows those channels to escape the ER and traffic to the sarcolemma. This was partially addressed by Marx et al. with experiments performed in a heterologous expression system, where they expressed WT channels and DHP-insensitive WT or DHP-insensitive AID mutant channels. Addition of nisoldipine resulted in remaining *I*_Ca_ in the DHP-insensitive WT case and almost eliminated *I*_Ca_ in the DHP-insensitive AID mutant channels. The conclusion was that Ca_V_β-less channels could not hitch a ride with WT channels in tsA cells. To unequivocally dispel the Ca_V_β-Ca_V_α_1c_ trafficking hypothesis, a transgenic mouse that expresses only AID mutant channels would be the gold standard. Nevertheless, this study also revealed that Ca_V_β-less channels are refractory to β-adrenergic stimulation and formed the prelude to the ground-breaking discovery that binding of the small Ras-like G protein Rad to Ca_V_β on the Ca_V_1.2 channel complex, partially inhibits channel activity until Rad it is phosphorylated by PKA, causing it to dislodge from Ca_V_β and revealing the enhanced open probability attributed to adrenergic regulation of these channels [39].

### 3.3. Ca_V_1.2 Recycling

Membrane protein expression is maintained in the face of ongoing endocytosis by both delivery via the biosynthetic pathway and from the endosomal recycling pathway (see Figure 2). Rab GTPases choreograph the trafficking of vesicular cargo from the membrane, through endocytosis, sorting, and subsequent recycling or degradation [65]. The >60-member family of Rab proteins is the largest family in the superfamily of Ras small GTPases. These proteins regulate the tethering or docking of vesicles to different membrane and endomembrane compartments, and may play roles in transport of vesicles along different cytoskeletal filaments by interacting with specific motor proteins [66]. For example, insulin reportedly stimulates Rab4 activity and association with the motor protein kinesin II (KIF3) to facilitate microtubule-mediated delivery and plasma membrane insertion of the GLUT4 glucose transporter in 3T3-L1 adipocytes [67]. Rab4 is known to facilitate so-called ‘fast recycling’ (t_1/2_ = 1–5 min) of vesicular cargo from early endosomes back to the plasma membrane [68,69]. Rab11 is another Rab protein which is known to facilitate ‘slow recycling’ (t_1/2_ = 12–30 min) of vesicular cargo from recycling endosomes back to the plasma membrane [68,69], and reportedly interacts with actin-filament associated myosin V motor proteins [70]. In the heart, the role of Rab protein interactions with cytoskeletal elements in Ca_V_1.2 channel recycling has not yet been investigated but our recent work suggests the presence of three endosomal reservoirs of Ca_V_1.2 channels located on: (1) Rab4-positive early endosomes, (2) Rab11-positive recycling endosomes, and (3) Rab7-positive late endo/lysosomes [54]. This recent work further provided the first reports of Ca_V_1.2 channel trafficking and recycling in live adult mouse ventricular myocytes [54]. This was achieved by transducing cardiomyocytes in vivo via retro-orbital injection with a cardiotropic AAV9-packaged, fluorescent protein-tagged Ca_V_β_2_ (AAV9-Ca_V_β_2_-paGFP) which essentially served as a fluorescent ‘biosensor’ to enable monitoring of the localization and dynamics of a portion of the endogenous channels. Total internal reflection fluorescence (TIRF) imaging using an ~150 nm evanescent field of excitation light, allowed examination of channel dynamics in the surface sarcolemma and initial portion of the t-tubular membrane in isolated, transduced cardiomyocytes, and revealed ongoing, rapid insertion and removal of channels [54,59] that likely reflects delivery from both the biosynthetic and recycling pathways, and counterbalancing endocytosis. Often these insertion and removal events involved entire clusters of channels although smaller discrete events that may represent removal of individual channels were also observed. The arrival and departure of channel clusters implies that Ca_V_1.2 channels can cluster in intracellular compartments. This idea was corroborated by immunostaining experiments that revealed clusters of channels on endosomes, and others lined-up in vesicles along microtubules. In agreement with the previously reported ~3 h lifetime of plasma membrane Ca_V_1.2 channels [19], 2 h cytoskeletal disruption with nocodazole, latrunculin A, or a combination thereof, produced no change in *I*_Ca_ density measured with whole-cell patch clamp electrophysiology, or in the overall expression of sarcolemmal channels assessed with super-resolution microscopy of immunostained channels. While this study did not explicitly examine the role of the cytoskeleton in ongoing (unstimulated) Ca_V_1.2 channel insertion and endocytosis, a previous study in tsA-201 cells using TIRF-fluorescence recovery after photobleaching (TIRF-FRAP) revealed a significant deficit in channel delivery to the membrane when microtubules, actin, or both were pharmacologically disrupted, and intracellular dynamics of vesicular Ca_V_1.2 was largely halted by these treatments [58]. With regards to the dependence of Ca_V_1.2 endosomal recycling on the cytoskeleton, in HL-1 cells Rab11a-mediated recycling of Ca_V_1.2 channels occurs along actin filaments whereas Rab4-dependent fast recycling is thought to occur along microtubules [71]. Thus, a model of Ca_V_1.2 channel recycling is beginning to emerge as illustrated in Figure 2.

### 3.4. Endocytosis and Retrograde Transport of Ca_V_1.2

There remains a distinct absence of studies on Ca_V_1.2 channel endocytosis in cardiomyocytes. As already discussed, channel removal from the sarcolemma has been visualized in these cells [54,59] but the mechanisms underlying it, and focused study of channel removal/endocytosis from distinct compartments of the membrane such as the t-tubules, crest, or caveolae, has not been explored. It is known that the endosomal recycling pathway is replenished by endocytosed channels, as nicely illustrated by the apparent increase in plasma membrane localized Ca_V_1.2, and concominant reduction of the Rab11-positive pool of endosomal Ca_V_1.2 channels when dynamin-dependent endocytosis was pharmacologically inhibited with dynasore in HL-1 cells [71]. Cell surface biotinylation experiments performed on adult ventricular myocytes also suggest that dynasore treatment increases the surface expression of Ca_V_1.2 [72]. However, dynasore is known to have off-target effects and can inhibit both dynamin-dependent and dynamin-independent endocytosis [73]. Like other membrane proteins, endocytosis of Ca_V_1.2 channels, may occur via a dynamin-dependent mechanism which may be further subclassified into either clathrin-dependent or clathrin-independent, or it can occur though dynamin-independent mechanisms [74,75]. In neurons, depolarization and activity dependent endocytosis of Ca_V_1.2 channels has been reported to occur and is thought to impart a level of protection against Ca^2+^ overload and associated neurotoxicity [76,77,78]. More in depth investigations are required to determine whether a similar cardioprotective mechanism occurs in cardiac muscle cells and to fully explore the mechanisms of endocytosis in these cells.

### 3.5. Ca_V_1.2 Degradation

Upon endocytosis, Ca_V_1.2 channels enter early endosomes (also known as sorting endosomes) where they are sorted and designated for either recycling or degradation [79]. Sorting signals, which enable recognition that a given cargo should be routed to the degradation pathway, include the presence of covalently attached ubiquitin [80]. Ubiquitination is a post-translational modification that involves addition of small 7 kDa ubiquitin to target proteins in a process that involves a series of enzymes called E1, E2, and E3 [80]. E1 activates ubiquitin in an ATP-dependent manner, and E2 then transfers it to E3-ubiquitin ligases which then covalently attach one or more ubiquitins to a lysine residue on their target protein. Ubiquitination of membrane proteins can stimulate their endocytosis, and/or function as a sorting signal recognized in the early endosome and result in targeting to late endosomes and lysosomes for degradation [81]. Ubiquitination can also occur to newly formed Ca_V_1.2 channels on the ER membrane and when it occurs there, channels are targeted to the proteasome for degradation. Figure 2 (top panel) shows an illustration of the endosomal degradation pathway, highlighting the role of Rab7 which regulates movement of vesicular cargo to late endosomes and lysosomes for degradation. Rab11b is also illustrated there in the recycling endosome where, it reportedly plays a role in targeting Ca_V_1.2 channels out of the recycling endosome and toward degradation in mouse neonatal cardiomyocytes [82]. This is in contrast to the role that we and others have reported for its close family member Rab11a in facilitating slow recycling of Ca_V_1.2 in HL-1 cells [71] and adult mouse ventricular myocytes [54].

Nedd4 ubiquitin ligases are expressed in the heart and have been reported to ubiquitinate various cardiac ion channels including Na_V_1.5 and hERG [80]. Nedd4-1 reportedly plays a role in reducing total and surface membrane expression of Ca_V_1.2 α_1c_ in transfected tsA-201 cells by promoting channel degradation [83]. Furthermore, co-expression of an adaptor protein called lipopolysaccharide-induced tumor necrosis factor (LITAF) was found to enhance α_1c_ ubiquitination levels [84]. In rabbit cardiomyocytes, overexpression of LITAF has been found to decrease I_Ca_, reduce Ca^2+^ transient amplitude, and lower total α_1c_ transcriptional expression [84]. An earlier study concluded that Nedd4-1 effects on Ca_V_1.2 were not direct as they could not detect ubiquitination of any of the channel subunits in transfected tsA-201 cells but did observe a significant reduction of I_Ca_, and surface biotinylation assays and western blots revealed reduced surface and total cellular channel expression [83]. Brefeldin-A, an inhibitor of ER-Golgi trafficking, abrogated the Nedd4-1 effects suggesting that the enzyme was acting to promote sorting of newly synthesized channels for degradation, even before their forward traffic to the membrane. Furthermore, Nedd4-1 dependent degradation of Ca_V_1.2 was prevented by MG132 an inhibitor of the proteasome, while lysosomal inhibitors also impacted the regulatory effects on Ca_V_1.2, implying that Nedd4-1 promoted channel sorting toward both of these degradation pathways. Interestingly, the Nedd4-1 effects on channel degradation were dependent on co-expression of Ca_V_β subunits. Recall that binding of Ca_V_β to Ca_V_α_1c_ in the ER, is thought to protect neuronal Ca_V_1.2 channels from ubiquitination and proteosomal degradation [52], so the dependence of this Nedd4-1 effect on Ca_V_β implies interference with its binding or chaperoning of α_1c_. A similar dependence on Ca_V_β was also noted in the more recent Moshal et al. study [84].

An elegant study from the Colecraft lab recently capitalized on the importance of Ca_V_β subunits for stable membrane expression of Ca_V_1.2 in their report on the creation of a potent engineered Ca_V_1.2 channel inhibitor which targets the catalytic domain of Nedd4-2 E3-ubiquitin ligase to Ca_V_β subunits using a nanobody [85]. Adenoviral transduction of adult guinea pig cardiomyocytes with this cleverly named ‘Ca_V_-aβlator’ led to complete eradication of I_Ca_, due to removal of Ca_V_1.2 from the dyad and routing to Rab7 positive late endosomes for degradation. Thus, despite the doubt cast on whether Ca_V_β plays an essential role in Ca_V_1.2 forward trafficking, it seems to play an important role in regulating its degradation and in this way controlling its membrane availability. Moreover, given that Ca_V_-aβlator was targeted toward the Ca_V_β subunit, yet ubiquitination was detected on both Ca_V_β and Ca_V_α_1c_, leading to channel redistribution from the dyad to Rab7 positive late endosomes, this work raises the intriguing question, does Ca_V_β binding to Ca_V_α_1c_ at the sarcolemma confer a protection from ubiquitination in an analogous manner to that described in the ER of neurons [52]? This question remains unanswered.

## 4. Ca_V_1.2 Localization and Targeting

### 4.1. Dyads

To fulfill their function as a channel for Ca^2+^ flux from the extracellular milieu into the cytosol, Ca_V_1.2 channels need to make their way to the sarcolemma. In the heart, the sarcolemma is a complex system of periodically arranged t-tubules approximately coincident with the z-lines, as well as the region on the surface of the cell between t-tubules known as the surface sarcolemma or crest [86,87]. T-tubules plunge 2–9 µm deep into the myocytes [87] and bring the membrane into close (~12 nm) proximity of junctional sarcoplasmic reticulum (jSR) localized ryanodine receptors (RyR2) at sites known as dyads or couplons. At some locations, the surface sarcolemma also comes into close apposition with the jSR and these non-t-tubular couplons are estimated to represent 22–25% of the total cellular couplon content [88]. Several reports have weighed in on the proportion of total Ca_V_1.2 expressed at dyads with Scriven et al. estimating that 75% of Ca_V_1.2 channel clusters reside at these specialized initiation sites of EC-coupling [89], while Pásek et al. found that approximately 80 % of Ca_V_1.2 is localized to the t-tubule membranes [90]. Dyadic Ca_V_1.2 channels are essential components of cardiac EC-coupling, this is demonstrated during heart failure when the architecture of cardiomyocytes is remodeled, and t-tubules become fractured, displaced, disorganized, and separated from the jSR [72] creating orphaned RYR2 [91], and resulting in reduced Ca^2+^ transient amplitude, contractile dysfunction, and promotion of arrhythmia promoting dyssynchronous Ca^2+^ release events [92,93,94,95]. β_2_-adrenergic receptors (β_2_-ARs) preferentially signal on the t-tubule membrane [96]. In contrast, β_1_-adrenergic receptors (β_1_-ARs) are located across the entire sarcolemma and produce less restrictive, global signals [96,97,98]. Regulation of Ca_V_1.2 channels by β-ARs is the most important regulatory pathway for tuning of cardiac EC-coupling to meet metabolic and hemodynamic demands.

Given the importance of t-tubule localized Ca_V_1.2 channels for the fundamental process and tuning of EC-coupling, it is vital that an efficient trafficking route exists to deliver and maintain a functional Ca_V_1.2 channel population at these sites. It has been determined that targeted transport of Ca_V_1.2 channels to the t-tubule membrane is conferred by Bridging integrator 1 (BIN1) [72], a member of the membrane-curvature mediating BAR (Bin1-Amphiphysin-Rvs) domain superfamily that is also involved in biogenesis and maintenance of the t-tubule network [99,100,101]. BIN1 has been shown to anchor microtubules at the t-tubule membrane, providing a delivery ‘hub’ for Ca_V_1.2 channels as they exit the TGN and travel in vesicles along these cellular highways in the anterograde trafficking pathway [72]. A multitude of evidence supports this idea as discussed below. Firstly, immunolabeling of Ca_V_1.2 and BIN1 in ventricular myocytes has revealed the two proteins colocalize along the t-tubules, biochemical studies have indicated they co-IP, while transfection-mediated overexpression of BIN1 in atrial HL-1 and non-cardiac HeLa cell-lines co-transfected with Ca_V_1.2 results in enhanced formation of membrane invaginations and surface expression of Ca_V_1.2 [72]. Lentiviral transduction of human embryonic stem cell-derived cardiomyocytes (hESC-CMs) with BIN1 facilitates development of the t-tubule network and Ca_V_1.2 clustering, cooperative gating, and overall activity (P_o_) [101]. Dynamic imaging of BIN1 and α-tubulin in HeLa cells has revealed tethering of microtubules at BIN1-positive sites [72]. In the more architecturally complex adult ventricular myocytes, vesicular Ca_V_1.2 has been shown to decorate cardiomyocyte and microtubules [54,72]. BIN1 knockdown in the heart either with siRNA [72] or shRNA [102], a cardiac specific knockout mouse model [99], or pathologically during heart failure [102], results in reduced Ca_V_1.2 channel expression at the cell surface and t-tubules [102]. Further evidence of a role for microtubules and BIN1 in Ca_V_1.2 delivery to the surface is provided by the reported blunting of cell surface accumulation of Ca_V_1.2 in cells where both dynamin-mediated endocytosis and microtubule-dependent delivery were pharmacologically disrupted with dynasore and nocodazole over 18–24 h [72]. This dependence on microtubules for maintenance of surface Ca_V_1.2 channel expression appears absent in the less specialized atrial HL-1 cell-line lacking in t-tubules wherein 18 hr incubation in nocodazole left Ca_V_1.2 channel *I*_Ca_ density unaltered [71].

The precise nature of the molecular interaction between BIN1 and microtubules has not yet been fully elucidated but may involve the cytoplasmic linker protein CLIP-170 [103,104]. Localization of phosphorylated CLIP-170 is most evident in the intercalated disks of adult mouse cardiomyocytes [105] but close inspection of the images in the aforementioned article reveals a robust periodic staining pattern that appears to coincide with the z-lines and so conceivable that CLIP-170 could be an intermediate between BIN1 and microtubules in these cells. However, in skeletal muscle this CLIP-170—BIN1 interaction appears to at least partially rely on the BAR domain of BIN1, as a point mutation in this region strongly reduced co-IP of BIN1 with CLIP-170 [103]. This is somewhat in conflict with a previous report that truncated BIN1, named BIN1-BAR (as it retains the BAR-domain important for the membrane curvature-mediating effects of BIN1, but lacks the coiled-coil and SRC homology 3 (SH3) domains,) fails to recruit Ca_V_1.2 channels to the surface membrane [72], the inference being that the BAR domain in the cardiac isoforms of BIN1 is neither necessary nor sufficient for the Ca_V_1.2 trafficking and targeting effects. It should be noted that these BIN1-BAR experiments were performed in HL-1 cells which, as already discussed above, may have a less robust reliance on microtubules for anterograde trafficking of Ca_V_1.2. It remains to be seen if BIN1 and CLIP-170 co-localize and co-IP in ventricular myocytes.

BIN1 has been reported to bind to another cytoskeletal highway, actin via its BAR domain [106]. The SH3 domain of BIN1 has further been found to interact with neuronal Wiskott–Aldrich syndrome protein (N-WASP) [107], and there is evidence that the cardiac-specific isoform of BIN1 (lacking exon 7, 11 and 14–16 but containing exons 13 and 17, A.K.A. BIN1 + 13 + 17), can bind to and activate N-WASP to promote actin polymerization by Arp2/3 complexes [99,108]. BIN1 + 13 + 17 also associates with F-actin and α-actinin, and these interactions likely stabilize t-tubules by anchoring them to the z-lines via α-actinin [99]. Indeed, actin stabilization with cytochalasin-D is well-known to preserve the t-tubule network in cultured cardiomyocytes [109,110]. Additionally, BIN1 + 13 + 17 is thought to be the main cardiac isoform responsible for generating micro-folds on the t-tubule membrane [99]. It has been speculated that these microfolds may limit the lateral diffusion of Ca_V_1.2 channels in the membrane and thus facilitating channel clustering. Indeed, in a conference abstract we have yet to develop into a full manuscript [111], we reported that Ca_V_1.2 channel clusters are ~42% smaller in cardiac-specific BIN1 heterozygous knockout (BIN1^+/−^) mouse ventricular myocytes that have a less dense population of t-tubule micro-folds than WT counterparts [99]. Pharmacological disruption of actin with latrunculin-A reportedly decreases the amount of membrane in ventricular myocyte t-tubules after 24 h in culture, compared to controls, suggesting that actin organizes and supports the highly folded regions of the t-tubules [99]. This finding appears contrary to a previous report that inhibition of actin polymerization with latrunculin-B enhances membrane tubule formation by F-BAR and BAR-proteins [112]. Two explanations were presented for this enhanced tubulation: firstly, actin disruption removes the stabilizing membrane scaffold and permits enhanced membrane deformation; second, they suggested an indirect antagonistic effect of the disruption of actin on dynamin such that endocytic vesicles that normally bud-off the membrane to internalize proteins and maintain homeostatic membrane protein populations, no longer underwent efficient fission and instead promoted elongation of membrane tubules. This cell biological study was not performed on cardiomyocytes but on more architecturally primitive COS-7 cells and did not specifically test the effect of actin disruption on BIN1-induced membrane tubulation, but focused on other BAR-domain proteins. In our recent work, we found that short-term (2 h) actin disruption with latrunculin-A did not appreciably change Ca_V_1.2 clustering, membrane expression, or current density under basal, unstimulated conditions [54].

Another class of cardiac ion channel, Cx43 hemichannels are known to undergo targeted delivery to the specialized cardiomyocyte membrane region of the intercalated disk in a manner that involves actin ‘rest-stops’ [113]. These are sites of pause for Cx43-containing vesicles that may have traveled from the TGN along a microtubule and are then handed-off to actin to form a pool of sub-membrane channels. Mobilized channels are ultimately handed-back to microtubules to be delivered to their final destination. These actin rest-stops may serve as sorting nexuses to redirect microtubule-mediated delivery to specialized sub-domains, and/or as pick-up locations to add accessory proteins, and/or as intracellular reservoirs to store ready-made channels which can be rapidly inserted into the membrane when demand arises. It remains to be determined whether actin rest stops play a role in Ca_V_1.2 channel trafficking in the heart but recent evidence from our group suggests that the mobilization of an internal pool of endosomal channels in adult mouse ventricular myocytes is affected by actin disruption pointing to a role for actin in the endosomal recycling pathway [54]. In HL-1 cells, actin disruption reportedly leads to impaired recycling of Ca_V_1.2 channels via the Rab11a pathway from recycling endosomes to the membrane [71]. A noteworthy point to consider when examining the role of actin in cardiomyocytes is that somewhat oddly, cytochalasin-D, a drug that is widely used as a means to disrupt actin polymerization, appears to have a stabilizing effect on actin in these cells, explaining why it preserves cell shape and prevents loss of t-tubules in culture [109,110]. Latrunculin-A on the other hand seems to act in the expected manner to disrupt the actin cytoskeleton [99,109]. Stabilization of actin with cytochalasin-D reportedly facilitates Ca_V_1.2 trafficking in cultured cardiomyocytes and prevents the peri-nuclear accumulation of the channels seen in cultured cells without cytochalasin-D supplementation [109]. This implies a role for actin in Ca_V_1.2 trafficking through the biosynthetic and anterograde trafficking pathway. However, actin is also known to play a role in stabilizing and guiding microtubules, so it may also be the case that stabilization of actin and preservation of cell shape and architecture, simply facilitates forward trafficking along microtubules [114,115,116]. Given the preponderance of data supporting the role of microtubules in forward trafficking of Ca_V_1.2, it seems likely that a substantial portion of the cytochalasin-D effect could be explained by this supporting role of actin.

### 4.2. Caveolae

Caveolae represent another specialized sarcolemmal compartment where a subset of Ca_V_1.2 channels are known to reside [117]. It has been estimated that as much as 15% of sarcolemmal Ca_V_1.2 reside in caveolae [35]. At ~50–100 nm in diameter [118], these flask shaped, cholesterol and sphingolipid rich invaginations of the sarcolemma are much smaller than the ~200–300 nm mean diameter t-tubules [119], the contrast in size between the two can be appreciated on freeze-fracture electron micrographs of rabbit ventricular myocytes [120]. Caveolae decorate the surface and t-tubular sarcolemma of cardiomyocytes although they are notably absent from dyadic regions [120]. This is perhaps a geometric phenomenon as the dyadic cleft is only ~12–15 nm wide to allow close proximity between the t-tubular Ca_V_1.2 and jSR localized RYR2, thus the physical restrictions of that narrow space intrinsically exclude 50–100 nm diameter caveolae. The precise role of caveolar Ca_V_1.2 channels in the heart remains unclear with some studies suggesting their involvement in EC-coupling [121], others finding no role in EC-coupling but supporting a role in ET-coupling [35], and others still finding no role in either physiological processes [122]. In terms of channel regulation, β_2_-AR are known to specifically associate with Ca_V_1.2 and Cav-3 in caveolae [117], and intact caveolae are necessary for β_2_-AR-mediated regulation of Ca_V_1.2 channels, but not for their regulation by β_1_-ARs. How the channels are targeted to these microdomains remains unknown. The fact that caveolae are sub-diffraction limit structures may have hampered their study thus far but the increased resolution afforded by ‘super-resolution’ light microscopy techniques may help provide answers as to how Ca_V_1.2 channels are localized to these specialized domains, and the role they play there.

## 5. Ca_V_1.2 Lifetime

Studies of the lifetime of Ca_V_1.2 channels in the t-tubule sarcolemma and the caveolar compartments would give us information about the dynamics of channel turnover and may reveal differences in delivery and removal mechanisms during health and disease, or circumstances that promote channel insertion or endocytosis. However, there are no reports of Ca_V_1.2 channel lifetime in adult cardiomyocytes, likely due to a lack of live cell fluorescent markers of the channel and the resistance of these cells to chemical transfection methods. Existing measurements of Ca_V_1.2 channel lifetime have all been performed in immortalized cell-lines, and none examine specific sarcolemmal sub-populations of channels. Nonetheless, there are some interesting insights to be gained from studies in heterologous expression systems. Pulse-chase experiments monitoring the percentage of ‘pulsed’ channels remaining in the membrane fraction up to 10 h post-chase, performed on transiently transfected human embryonic kidney cells (HEK293) expressing the pore forming α_1c_ and auxiliary β_2a_ subunits suggest the channels have a half-life of ~3 h [19]. A different study in HEK293T cells tracked pulse-chased channels over a 25 h period after the chase and reported a half-life of α_1c_ of ~25 h, after an initial rapid degradation in the first 4 h post-chase [123]. The difference in these results is likely because the first study was examining the membrane fraction while the second examined total cellular Ca_V_1.2. Altogether, these results suggest that membrane Ca_V_1.2 turns over more rapidly than cellular Ca_V_1.2. In agreement with that, a more recent study found that while endogenous *I*_Ca_ current density in HL-1 cells was maintained at a stable level for >18 h despite disruption of microtubule-based channel delivery with nocodazole, internalization of transfected Ca_V_1.2-HA channels labeled with a 10 min pulse of anti-HA DyLight 488, was seen to occur a much faster rate with a time constant of internalization of 7–8 min [71]. A similar 9–10 min time constant was obtained in tsA201 cells using an approach in which photoactivatable GFP tagged Ca_V_1.2 was photoactivated and its presence at the membrane measured over the subsequent 100 min [124]. Conrad et al. posited that if the membrane population can remain at a stable expression level for >18 h despite ongoing internalization of channels at this rapid rate, then there must also be an ongoing insertion of channels to counterbalance this [71]. They used a clever ‘double pulse-chase’ protocol to visualize this dynamic insertion of channels wherein initial surface Ca_V_1.2-HA were labeled with anti-HA DyLight 488, and 20 min later, a second pulse of anti-HA DyLight 561 revealed robust staining reflecting the presence of channels that were newly inserted during the intervening 20 min. The authors further concluded that surface membrane Ca_V_1.2 channel population in HL-1 cells is constantly and dynamically maintained by endosomal recycling. This endosomal recycling pathway was studied in more detail by our group in live adult mouse ventricular myocytes, where we found that a pool of readily insertable Ca_V_1.2 form a reservoir of channels, which is replenished by channel internalization, and can be rapidly mobilized to the t-tubule sarcolemma in times of acute stress [54,59].

## 6. GPCR Regulation of Ca_V_1.2 Trafficking

### 6.1. Stimulated Insertion

**Figure 3:**
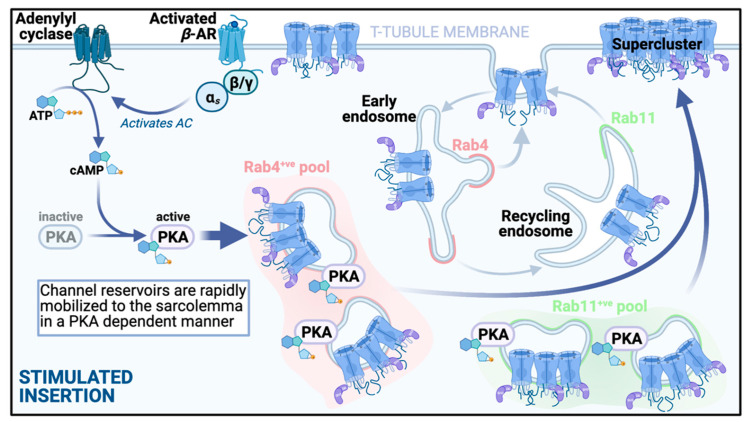
β-adrenergic receptor stimulated insertion. An illustration of the stimulated insertion pathway that ensues upon acute activation of G_s_-coupled β-ARs. Reservoirs of preformed channels are present in subsarcolemmal pools. Activation of the β-AR/AC/cAMP/PKA pathway triggers rapid mobilization of these individual and clustered channels to the t-tubule membrane, along Rab4 and Rab11 dependent trafficking pathways. As a result of this enhanced insertion, there is an increase in the number of functional channels at the sarcolemmal. Large superclusters of Ca_V_1.2 channels form on the t-tubule membrane. These channels exhibit more cooperative interactions and generate enhanced Ca^2+^ influx to amplify EC-coupling [59].

The presence of several internal pools of ready-made Ca_V_1.2 channels, invites the thought that perhaps there is some redundancy in these reservoirs under steady-state basal conditions, and that additional channels from the pools could be rapidly mobilized to the sarcolemma to increase channel and current density in times of high demand. Our lab recently reported that activation of β-ARs with isoproterenol (ISO; 100 nM) in isolated adult mouse ventricular myocytes, triggers PKA-dependent augmentation of sarcolemmal insertion of Ca_V_1.2 channels [54,59]. We visualized this dynamic process using the aforementioned AAV9-Ca_V_β_2_-paGFP biosensor approach, and found that the stimulated insertions occurred extremely rapidly, with a τ = 4.12 s at physiological temperature [54]. Immunostaining experiments revealed that these inserted channels were mobilized from subsarcolemmal pools of Rab4 positive endosomes and Rab11 positive recycling endosomes (see illustration of the pathway in Figure 3). This manifested as significantly reduced colocalization between these specific endosomal markers and Ca_V_1.2 after ISO, suggesting that the Ca_V_1.2 cargo of these endosomes had been delivered to the sarcolemma. The role of the Rab4-dependent fast recycling pathway, and the Rab11-dependent slow recycling pathway was confirmed by experiments in transiently transfected tsA-201 cells using dominant negative (GDP-locked) and constitutively active (GTP-locked) Rab4 and Rab11 to study the impact on ISO-induced increase in membrane Ca_V_1.2 channel expression. Furthermore, super-resolution microscopy of the t-tubule and sarcolemmal crest regions revealed that stimulated channel insertions occurred predominantly at the t-tubule membrane. This has implications for EC-coupling as more channels in the t-tubule dyad regions could enhance EC-coupling. Indeed we found that the larger superclusters of Ca_V_1.2 channels observed in response to ISO promoted enhanced cooperative gating behaviors [59]. This gating behavior is a known property of Ca_V_1.2 channels in which physically interacting channels within a cluster can communicate with one another via Ca^2+^-calmodulin dependent associations between their C-terminal tails [56]. The opening of the highest activity channel in the cluster drives the other attached channels leading to amplification of Ca^2+^ influx [55]. This raises an interesting point when one considers that ISO-stimulated insertion of channels is a PKA-dependent phenomenon, wherein super-clustering and enhanced sarcolemmal expression of Ca_V_1.2 is prevented by pharmacological inhibition of PKA with PKAi or H-89 [59]. It is unknown whether PKA-phosphorylation of Ca_V_1.2 channels can occur while they are localized on endosomal membranes but there is a PKA-anchoring protein called D-AKAP2, that associates with endosomes in cardiomyocytes and displays an ISO-stimulated enhanced colocalization with Rab11 positive endosomes, that could potentially support that [54]. Notably, D-AKAP2 has been shown to regulate transferrin receptor recycling through interactions with Rab4 and Rab11 [125], and a human functional polymorphism in D-AKAP2 (1646V), is known to lower heart rate variability [126], suggestive of a heart that cannot respond well to stressors. PKA-phosphorylation of the Ca_V_1.2 channel complex leads to enhanced open probability (P_o_) of these channels, and increased longer-lived mode 2 openings, that generates enhanced Ca^2+^ influx and the positive inotropic response downstream of β-AR activation during the fight-or-flight response [39,127,128,129]. Thus, it is possible that just a small number of high P_o_, phosphorylated channels inserted into the membrane from the endosomal pool could have a disproportionately large effect on *I_Ca_* and EC-coupling. This idea that β-AR signaling-mediated regulation of Ca_V_1.2 channel recycling could by itself, generate the stereotypical augmentation of *I*_Ca_ seen in cardiomyocytes during flight-or-flight, is supported by functional patch clamp data in which disruption of endosomal channel insertion with cytoskeletal disruptors, abrogates the left-ward shift in voltage-dependent activation and enhanced *I*_Ca_ response to ISO despite preserved ISO-stimulated cAMP production and robust adrenergic signaling [54]. The idea that an increase in the number of functional channels in the sarcolemma could at least partially underlie the augmented *I*_Ca_ associated with β-AR signaling was previously suggested by Bean et al. in a 1984 study on frog ventricular heart cells where fluctuation analysis of *I*_Ca_ recordings revealed an ISO-stimulated increase in the number of functional channels per cell [130], although the same group later clarified that an increase in the number of functional channels did not necessarily mean there were more channels in the membrane, but could instead reflect previously quiescent channels that became more compelled to open in the presence of ISO [131]. Nonetheless, receptor stimulation of ion channel insertion from intracellular pools has been reported in several other cells and tissues including: (1) neurons where β_2_-AR signaling via G_αs_/adenylyl cyclase/cAMP/PKA stimulates Rab11-dependent insertion of AMPA receptors from recycling endosomes into the plasma membrane of dendritic spines [132]; (2) kidney, where V2R vasopressin receptor signaling through G_αs_/adenylyl cyclase/cAMP/PKA stimulates PKA-dependent insertion of aquaporin 2 (AQP2) from Rab11-positive recycling endosomes into the apical membrane [133,134]; and (3) heart, where two other cardiac ion channels, namely K_ATP_ and KCNQ1 are mobilized from Rab11-positive endosomal pools to the sarcolemma in response to acute stress [135,136,137].

### 6.2. Stimulated Endocytosis

**Figure 4:**
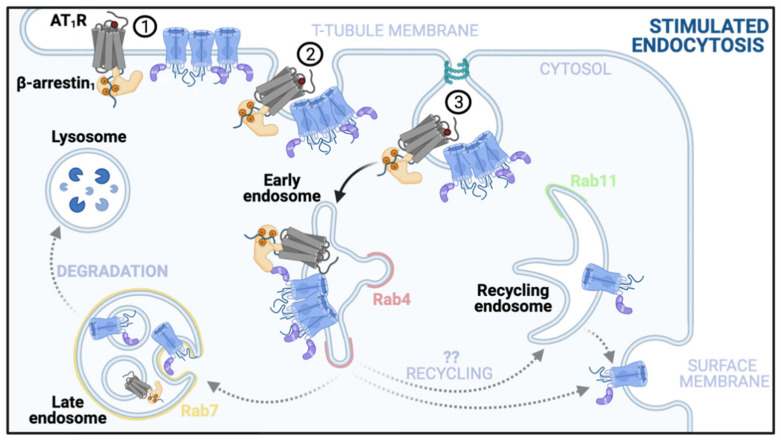
Angiotensin type 1 receptor stimulated endocytosis. Prolonged exposure to angiotensin II, over the course of an hour, leads to: (1) recruitment of β-arrestin_1_ to t-tubule localized Ca_V_1.2 channel-AT_1_R receptor complexes; (2) clathrin-coated pit formation, and (3) endocytosis. This reduces Ca_V_1.2 channel expression and I_Ca_. The identity of the cellular compartment to which the channels are targeted upon internalization are unknown as represented by the grey dotted arrows. Their fate may lie in lysosomal degradation or they may be stored in intracellular reservoirs, or finally, as indicated by the study by Altier et al., some channels may be recycled to other membrane regions, including the surface membrane which appeared to grow more intensely positive for Ca_V_1.2 immunostaining as t-tubular and dyadic expression dropped [138].

The endosomal reservoir of channels, which is constantly replenished by ongoing channel internalization, may similarly have the capacity to accommodate more channels if there is a need to reduce sarcolemmal channel density. This may occur if an analogous Ca^2+^ overload-preventative channel internalization system as has been described in neurons, also exists in cardiomyocytes [76,77,78]. Prolonged activation of AT_1_R receptors with angiotensin II has been reported to stimulate endocytosis/internalization of Ca_V_1.2 channels in adult rat cardiomyocytes [138]. This process is seen to occur over about an hour and involves β-arrestin_1_ recruitment to t-tubular Ca_V_1.2 channels. A preferential internalization of t-tubule localized channels occurs over the period of around an hour and leads to reduced *I_Ca_* and reduced amplitude Ca^2+^ transients and cell-shortening. It remains to be determined what endosomal pool these channels are targeted toward and whether they can be quickly recycled back to the membrane again after the angiotensin II stimulus is removed, or if they are targeted for degradation. Results from Hermosilla et al. suggest that a subpopulation of the endocytosed channels may be redistributed to surface membrane locations as indicated by enhanced immunostaining of Ca_V_1.2 on the surface membrane while t-tubular signal faded. This stimulated endocytosis pathway is represented in Figure 4.

Given the findings that β-AR stimulation can trigger rapid Ca_V_1.2 channel insertion into the t-tubule membrane [54,59], an obvious question to explore is whether subsequent phosphatase mediated dephosphorylation of Ca_V_1.2 can trigger internalization. This has been reported to occur in dendritic spines where, as already discussed above, a pool of intracellular GluA1-containing AMPARs is thought to transition to a readily insertable state upon phosphorylation AMPA receptors by PKA and/or calcium/calmodulin-dependent protein kinase II (CaMKII), or protein kinase C (PKC) [139,140,141]. The resulting plasma membrane insertion of the receptors, facilitates long term potentiation (LTP) by increasing channel open probability (*P*_o_) and homeostatic scaling up. Conversely, dephosphorylation by calcineurin (CaN) leads to receptor internalization, long term depression and homeostatic scaling down [142]. Whether a similar system exists in cardiomyocytes will make for an interesting future study.

## 7. Conclusions

Ca_V_1.2 channel expression on the cardiomyocyte sarcolemma is tightly controlled and regulated. They are constantly being internalized, sorted, recycled, degraded, but at steady-state this complex system reaches an equilibrium where a constant level of expression is maintained. Regulatory mechanisms have emerged in recent years that suggest the balance between channel insertion and internalization can be tilted by GPCR-mediated regulatory pathways. Rapid channel insertion from endosomal pools provides extra Ca_V_1.2 channels to the t-tubule membrane during fight-or-flight to fuel the harder-working myocytes with more Ca^2+^ influx capacity to generate a positive inotropic effect and meet the enhanced hemodynamic and metabolic demands associated with this response. Furthermore, prolonged AT_1_R signaling triggers channel endocytosis and reduced expression generating a negative inotropic effect. We await further detailed characterization of the kinetics of Ca_V_1.2 channel trafficking, and of when in the anterograde pathway the auxiliary subunits join the complex. Moreover, the specific mechanism of targeting and transport of these channels to non-t-tubular locations including caveolae and the surface membrane remains to be determined. An interesting avenue for future research in those topics is that of the role of the junctional tethering protein junctophilin 2 (JPH2) in targeting Ca_V_1.2 to microdomains within the sarcolemma. A recent study reported that JPH2 recruits Ca_V_1.2 to lipid rafts on the T-tubules wherein overexpression of JPH2 in cultured rat cardiomyocytes led to increased channel density at the t-tubule and surface membrane that was not accompanied by an increase in total cellular Ca_V_1.2 protein, implying JPH2 increased channel trafficking rather than altered channel biosynthesis [143]. Future studies should also parse out the mechanistic details of trafficking alterations that may explain how failing hearts redistribute Ca_V_1.2 from the t-tubular sarcolemma to the surface membrane [144,145,146], and others that mediate the enhanced Ca_V_1.2 sarcolemmal expression observed with aging [147,148,149].

It will be important for the field moving forward to have a definitive measure of Ca_V_1.2 channel lifetime in cardiomyocytes and a full picture of how channels are targeted to caveolae, t-tubules, or the surface sarcolemma, and indeed how these targeting mechanisms become altered in disease. The recently developed Retention Using Selective Hooks (RUSH) system [150] may make more accurate measurements and visualization of Ca_V_1.2 channel trafficking feasible. This system allows the user to trap channels at certain points along the biosynthetic or recycling pathways, release them at will, and observe the kinetics and direction of the trafficking of the channels as they travel to their destinations in the cell. The RUSH system utilizes various protein ‘hooks’ fused to streptavidin which reversibly anchor and retain streptavidin-binding peptide (SBP) fused proteins of interest in a cellular compartment. The addition of biotin outcompetes the SBP/streptavidin interaction and severs the connection between the hook and bait allowing the protein to be released into the secretory pathway. This system has recently been used to study glutamate receptor [151], KCNQ1/KCNE1 [152], and to study altered transport kinetics during pathological conditions [153]. One could envisage that this system could be used to determine when and where the various auxiliary subunits of the channel join the α1 subunit.

In addition, future studies should examine size and mobility of the endosomal pool of channels and how it may be altered by aging or disease. Furthermore, as already mentioned above, we eagerly anticipate future determination of the molecular mechanisms underlying PKA-triggered mobilization of the endosomal channel reservoirs and whether the inverse effect is seen with phosphatase-mediated channel dephosphorylation. In addition, the question still remains open as to whether the triggered insertion of Ca_V_1.2 channels downstream of β-adrenergic receptor stimulation, is accompanied by an increase in RyR2 expression on the other side of the dyad. There is some evidence that this might be the case as cardiomyocyte stimulation with isoproterenol reportedly enhances phosphorylated RyR2 clustering in dyadic regions [154]. A coordinated stimulated enhancement of both Ca_V_1.2 and RyR2 in dyadic regions during fight-or-flight could facilitate an even larger inotropic response.

This relationship between Ca_V_1.2 and RyR2 expression raises a final intriguing idea that should be explored. Recent work has suggested that the functional expression of ion channel complexes at the cardiomyocyte sarcolemma is regulated by a ‘microtranslatome’ whereby mRNA transcripts of Na_V_1.5 and hERG associate with each other during translation to coordinate and regulate the balance of expression of these channels [155]. A fine balance between the depolarizing Na_V_1.5 and repolarizing hERG channels is critical to maintain action potential production of the correct duration and to avoid arrhythmias. The idea of co-translational regulation of Ca_V_1.2 is an interesting one considering that gain-of-function mutations in Ca_V_1.2 can lead to long QT8 (Timothy syndrome) [5] and that aberrant interactions between adjacent Ca_V_1.2 channels can also facilitate arrhythmogenic activity [56,156,157]. Could there be association between Ca_V_1.2 channel transcripts that regulates its functional expression or balances it against a repolarizing channel? Could Ca_V_1.2 channel subunit transcripts associate and undergo co-translation that facilitates ER export? These are all open questions in this still developing field of Ca_V_1.2 trafficking regulation.
